# BBB-Permeable, Neuroprotective, and Neurotrophic Polysaccharide, Midi-GAGR

**DOI:** 10.1371/journal.pone.0149715

**Published:** 2016-03-03

**Authors:** Vishruti Makani, Yong-gil Jang, Kevin Christopher, Wesley Judy, Jacob Eckstein, Kenneth Hensley, Nicolas Chiaia, Dong-Shik Kim, Joshua Park

**Affiliations:** 1 Department of Neurosciences, College of Medicine and Life Sciences, University of Toledo, Toledo, Ohio, United States of America; 2 Department of Pathology, College of Medicine and Life Sciences, University of Toledo, Toledo, Ohio, United States of America; 3 Department of Chemical Engineering, College of Engineering, University of Toledo, Toledo, Ohio, United States of America; Sungkyunkwan University, REPUBLIC OF KOREA

## Abstract

An enormous amount of efforts have been poured to find an effective therapeutic agent for the treatment of neurodegenerative diseases including Alzheimer’s disease (AD). Among those, neurotrophic peptides that regenerate neuronal structures and increase neuron survival show a promise in slowing neurodegeneration. However, the short plasma half-life and poor blood-brain-barrier (BBB)-permeability of neurotrophic peptides limit their *in vivo* efficacy. Thus, an alternative neurotrophic agent that has longer plasma half-life and better BBB-permeability has been sought for. Based on the recent findings of neuroprotective polysaccharides, we searched for a BBB-permeable neuroprotective polysaccharide among natural polysaccharides that are approved for human use. Then, we discovered midi-GAGR, a BBB-permeable, long plasma half-life, strong neuroprotective and neurotrophic polysaccharide. Midi-GAGR is a 4.7kD cleavage product of low acyl gellan gum that is approved by FDA for human use. Midi-GAGR protected rodent cortical neurons not only from the pathological concentrations of co-/post-treated free reactive radicals and Aβ_42_ peptide but also from activated microglial cells. Moreover, midi-GAGR showed a good neurotrophic effect; it enhanced neurite outgrowth and increased phosphorylated cAMP-responsive element binding protein (pCREB) in the nuclei of primary cortical neurons. Furthermore, intra-nasally administered midi-GAGR penetrated the BBB and exerted its neurotrophic effect inside the brain for 24 h after one-time administration. Midi-GAGR appears to activate fibroblast growth factor receptor 1 (FGFR1) and its downstream neurotrophic signaling pathway for neuroprotection and CREB activation. Additionally, 14-day intranasal administration of midi-GAGR not only increased neuronal activity markers but also decreased hyperphosphorylated tau, a precursor of neurofibrillary tangle, in the brains of the AD mouse model, 3xTg-AD. Taken together, midi-GAGR with good BBB-permeability, long plasma half-life, and strong neuroprotective and neurotrophic effects has a great therapeutic potential for the treatment of neurodegenerative diseases, especially AD.

## Introduction

Conventional treatments for neurodegenerative diseases address only symptoms without disease-modifying effect but with serious side effects [1–6]. Currently, there is no effective treatment for neurodegenerative diseases. As aged population grows very fast, the incidence of aging-related neurodegenerative diseases and their healthcare costs are increased exponentially. AD alone affects over 5 million people in the US and costs the US 100 billion dollars per year [7, 8]. Thus, it is of utmost urgency to find an effective treatment for neurodegenerative diseases.

Pharmacological inhibitors that are purposed to reduce pathogenic factors have been unsuccessful in exerting a disease-modifying effect [9–12]. Conversely, neurotrophic treatment that revives neurons and rebuilds synapses and neurites shows a promise in slowing neurodegeneration [8, 13–23]. Moreover, neurotrophic treatment appears to have a larger intervention window than preventive toxin-clearing approaches [24]. Thus, various neurotrophic peptides were tested regarding their efficacies in treating neurodegenerative diseases [8, 13–21, 23, 25, 26]. Brain-derived neurotrophic factor (BDNF) is one of the major targets for neurotrophic treatment [27, 28]. However, the poor BBB-permeability and short plasma half-life of neurotrophic peptides including BDNF lower their efficacy [29–33]. To overcome the limitations, viral vectors and mesenchymal stem cells that constantly produce neurotrophic peptides have been injected into the brain [34–36]. However, the invasiveness of surgical delivery, mutagenesis, and unregulated peptide production are of concern. Nanoparticles also have been tested for the intranasal delivery of neurotrophic peptide into the brain while the short plasma half-life of peptide is still a limiting factor [37–39].

**Fig 1:**
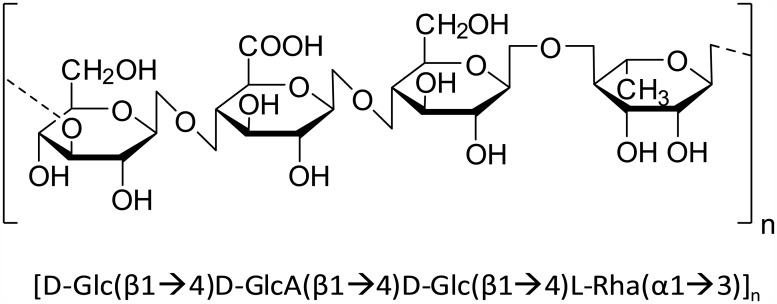
Repeating tetrasaccharide unit of low acyl gellan gum. Low acyl gellan gum consists of the repeating tetrasaccharide unit ([D-Glc(β1→4)D-GlcA(β1→4)D-Glc(β1→4)L-Rha(α1→3)]_n_) of low acyl gellan gum.

Recently, a group of polysaccharides were found to have neuroprotective effects [40–43], raising the possibility of using the polysaccharides for the treatment of neurodegenerative diseases. If the polysaccharides can penetrate the BBB, those are expected to exert longer physiological effect than peptides *in vivo* as polysaccharides generally have long plasma half-lives [44–47]. Among the polysaccharides, however, only chitosan shows BBB-permeability [37–39, 48, 49]. All these indications prompted us to search for a BBB-permeable and neuroprotective polysaccharide among natural polysaccharides that are approved by FDA for human use. Then, we discovered a BBB-permeable, long plasma half-life, neurotrophic, and neuroprotective polysaccharide, midi-GAGR, that is a 4.7kD cleavage product of low acyl gellan gum. Low acyl gellan gum is registered as ‘Everything Added to Food in the United States (EAFUS)’ (FDA 21 CFR 172.665). Low acyl (**LA**) gellan gum consists of a repeating tetrasaccharide, D-**G**lc(β1→4)D-Glc**A**(β1→4)D-**G**lc(β1→4)L-**R**ha(α1→3) (called ‘**LA-GAGR**’ in our study, Fig 1). LA-GAGR has few side effects in human at >160 mg/kg/day [50] and in animal at >1,000 mg/kg/day according to FDA report.

In addition, we found that midi-GAGR bound to FGFR1 and activated FGFR1-mediated neurotrophic signaling pathway [51–53]. FGFR1 is predominantly expressed in neurons including hippocampal and dentate gyrus neurons [54, 55] and contributes to neurite outgrowth, neuronal migration, and axonal pathfinding [56, 57]. Fibroblast growth factor 2 (FGF2), a major ligand for FGFR1 [58, 59], shows a good therapeutic potential for the treatment of neurodegenerative diseases. FGF2 enhanced survival and neurite outgrowth in hippocampal neurons *in vitro* [60, 61]. In animals, FGF2 reduced AD pathogenesis [62–65], improved memory [66–68], and reduced β-secretase and Aβ peptide [69, 70]. Nonetheless, the short plasma half-life and poor BBB-permeability of FGF2 lower its *in vivo* efficacy [67, 71]. FGFR1 can be also activated by its interaction with neural cell adhesion molecules (NCAMs) [72, 73]. Homodimeric NCAM180s interact with FGFR1 via its fibronectin type III (FN3) modules I and II and activate FGFR1 [74]. This interaction leads to the activation of two signaling pathways under FGFR1: (i) FGFR substrate 2a (FRS2a)-Shc-growth factor receptor bound protein 2 (Grb2)-protein kinase C (PKC)-Raf-mitogen activated protein kinase (MAPK)/extracellularly regulated kinase (ERK) kinase (MEK) pathway and (ii) phosphoinositide 3-kinase (PI3K)-Akt/phospholiase Cγ(PLCγ)-Ca^2+^-calmodulin kinase II (CaMKII) pathway [73, 75, 76]. Under NCAM180, fyn-focal adhesion kinase (FAK)-src-MEK-ERK pathway is activated [73]. ERK and Akt, then, phosphorylate CREB [77]. Recently, Fibroblast Growth Loop (FGL) peptide containing 15 amino acids in the 2nd FN domain of NCAM [74] was found to bind to FGFR1 and exert FGF-like effects [78]. FGL activates FGFR1 independently of NCAM [79]. FGL enhanced neuritogenesis and synaptogenesis, protected neurons from Aβ_42_ and 6-hydroxydopamine, improved learning and memory and synaptic transmission [52, 79–83]. In addition, Akt activated by FGL phosphorylates and inhibits glycogen synthase kinase 3β (GSK3β) [82], the kinase responsible for the hyperphosphorylation of tau (p-tau) and the formation of neurofibrillary tangles (NFTs) [84]. Although FGL shows some BBB-permeability [81] and 4-h plasma half-life [85], its peptide-ness is still a limiting factor.

Here, we present a novel alternative (midi-GAGR) to neurotrophic peptide that shows neuroprotective and neurotrophic effects, good BBB-permeability and long plasma half-life (~24 h). Our study will present solid evidence that supports the great potential of midi-GAGR for the treatment of neurodegenerative diseases.

## Materials and Methods

### Animals

Embryos (at the embryonic day of 17 [E17]) from female pregnant mice (BALB/C, Charles River Laboratories International Inc., Wilmington, MA) and female pregnant rats (E17, Sprague Dawley [SD], bred-in-house) were used to isolate primary cortical neurons for *in vitro* primary culturing. Adult female SD rats (12–16 weeks old, bred-in-house) were used for *in vivo* studies to examine the BBB-permeability and neurotrophic effect of midi-GAGR. 12-month-old 3xTg-AD mice (female, B6; 129-Psen1tm1Mpm Tg [APPSwe, tauP301L] 1Lfa/Mmjax, Jackson Laboratory, Bar Harbor, ME) were used for the studies to examine the effects of midi-GAGR on neurotrophic and neurodegenerative markers in 3xTg-AD mouse brains. Animals were housed at room temperature under a 12-h light/dark cycle. Food and water were provided *ad libitum*. All experiments were performed during the light phase (7 am-7 pm). All the procedures of animal use described in this study were approved by the Institutional Animal Care and Use Committee (IACUC) of University of Toledo College of Medicine and Life Science in accordance with National Institutes of Health guidelines.

### Antibodies

Antibodies to FGFR1 (SAB4300488), neurofilament 200 (NF200, N4142), and α-tubulin (T9026), synaptophysin (S5768), and βIII-tubulin (T2200) were purchased from Sigma (St. Louis, MO). Antibodies to PSD95 (sc-32290), pCREB (P-Ser133, sc-7978), CREB (sc-377154), glyceraldehyde-3-phosphate dehydrogenase (GAPDH, sc-32233), and growth associated protein 43 (GAP-43, sc-17790) were purchased from Santa Cruz Biotechnology, Inc. (Santa Cruz, CA). Antibody to PHF-tau (P-Ser202, AT8, MN1020B) was purchased from Thermo Scientific (Rockford, IL). Antibodies to Iba1 (ab5076) and pCREB (P-Ser133, ab30651) were purchased from Abcam Inc. (Cambridge, MA). Antibody to tau (610672) was purchased from BD Transduction Laboratories (Lexington, KY).

### Hydrolysis of low acyl gellan gum

1 mL of 1% salicin solution (1 g salicin [Sigma] in 100 mL of 0.1 M acetate buffer [pH 5]) was pre-warmed at 37°C for 6–8 minutes and mixed with 2 mg of α(1→3)-glycosidase (Sigma) to make the enzyme solution for the hydrolysis of low acyl gellan gum (LA-GAGR, CPKelco Co. [Atlanta, GA]). The enzyme solution was diluted to 0.1 mg/mL before use. 0.48 g of LA-GAGR was dissolved in 80 mL of the acetate/salicin solution. 8 mL of LA-GAGR solution was mixed with 2 mL of the enzyme solution in 15 mL polypropylene cornical tubes and incubated at 37°C (80 rpm) for 24, 48, or 72 h for the enzymatic digestion of LA-GAGR. Enzyme reaction was stopped by incubation in a hot water bath for 5 min and dried in a vacuum dryer (-60 cm Hg gauge) at 70°C. Dried gel pellet was then washed extensively in de-ionized water by stirring for 48 h (fresh water replaced every 12 h) to wash off salts and enzyme from the pellet and processed for viscosity measurement using a parallel plate rheometer (PPR, Rheometrics, Inc., Piscataway, NJ) equipped with rheometer software, TA Orchestrator (TA Instrument, Inc., New Castle, DE). From the viscosity-storage modulus profiles, the MW distributions of the cleavage products were determined using the RheoAnalyzer program (TomCoat Oy, Inc., Finland). The pellet was dissolved in 100 mL de-ionized water, autoclaved at 120°C for 45 min, aliquoted, and kept at -80°C until use.

### Drug treatment of Neuro2A (N2A) cells

N2A cells (passage 7–10, a gift from Dr. Marthe Howard at University of Toledo [ATCC^®^ CCL-131^™^]) were sparsely seeded on coverslips in Dulbecco’s modified Eagle’s medium (DMEM, Life Technologies, Grand Island, NY) containing 10% heat-inactivated fetal bovine serum (FBS), 5 g/L D-glucose, 110 mg/L sodium pyruvate, and 1 x Pen Strep (Life Technologies) and incubated at 37°C in a humidified 5% CO_2_ incubator for 48 h. Then, serum-containing medium was replaced with serum-free medium containing vehicle (H_2_O) or different concentrations of midi-GAGR (0.001, 0.01, 0.1, 1, and 10 μM) and incubated for 3 days prior to immunocytochemistry using anti-α-tubulin antibody followed by secondary Alexa_488nm_ antibody (Life Technologies) [86]. Coverslips were then mounted on glass slides using Fluoromount G (Fisher Scientific, Pittsburgh, PA). The images of cells were taken using a TCS SP5 multi-photon laser scanning confocal microscope (Leica Microsystems, Bannockburn, IL). The confocal microscope is equipped with conventional solid state, a ti-sapphire tunable multi-photon laser (Coherent, Santa Clara, CA), and acousto optical beam splitter AOBS. Images were taken with either 40 x or 20 x Zeiss alpha plan fluor oil objective (1.4 NA). Cells having the neurite length longer than 2 times the diameter of cell body were chosen for image analysis. To examine the protective effect of midi-GAGR against oxidative stress-induced neurite atrophy, differentiated N2A cells were pre-treated with midi-GAGR (0, 0.001, 0.01, 0.1, 1, and 10 μM) and then with either 4-hydroxynonenal (4HNE) (Cayman Chemical, Ann Arbor, MI) or H_2_O_2_ (Sigma). First, we determined the toxic dose ranges of 4HNE and H_2_O_2_ that cause neurite atrophy by treating differentiated N2A cells with 0, 1, 5, 10 or 25 μM of 4HNE for 48 h or 0, 1, 10, 50, 100, or 200 μM of H_2_O_2_ for 24 h. Then, the doses that showed maximum inhibitory effects were used to treat differentiated N2A cells along with 0, 0.001, 0.01, 0.1, 1, and 10 μM of midi-GAGR. The total neurite lengths of N2A cells in different conditions were measured using ‘Metamorph’ software (Molecular Devices, Sunnyvale, CA) and used to calculate average total neurite lengths.

### Drug treatment of primary rodent cortical neurons

We examined the protective effect of polysaccharides on primary cortical neurons from 4HNE, H_2_O_2_, and Aβ_42_ peptide (Sigma) using LIVE/DEAD^®^ Viability/Cytotoxicity Assay Kit (Life Technologies). Female pregnant mice (BALB/C, E17, Charles River Laboratories International, Inc.) or female pregnant SD rats (E17, bred in house) were anesthetized and dissected to obtain 8–9 embryos per animal. Cortical neurons were isolated from embryonic brains and differentiated on poly-L-lysine-coated coverslips in B27/neurobasal medium. For drug treatment before free radical treatment, mouse cortical neurons (5 days *in vitro* [DIV5]) were treated with vehicle (H_2_O), 1 μM of 5kD dextran (Sigma), alginate (Sigma), midi-GAGR, 0.01 μM of high acyl gellan gum (HA-GAGR, CPKelco Co.), or 0.1 μM of LA-GAGR for 24 h and then treated with 10 μM 4HNE or 50 μM H_2_O_2_ for 24 h, or 2 μM Aβ_42_ peptide for 48 h prior to viability/cytotoxicity assay. For drug co-treatment with free radicals, rat cortical neurons (DIV5) were treated with 1 μM of dextran, alginate, or midi-GAGR, 0.01 μM of HA-GAGR, or 0.1 μM of LA-GAGR along with either 10 μM 4HNE (24 h) or 2 μM Aβ_42_ peptide (48 h). To examine the extent to which midi-GAGR-mediated neuroprotection depends on FGFR1, rat cortical neurons (E17, DIV5) were pre-treated with 4 μM SU5402 (Sigma) for 6 h and treated with 10 μM 4HNE and either vehicle or 1 μM midi-GAGR for 24 h prior to cell viability/cytotoxicity assay. As controls, neurons were treated with SU5402, midi-GAGR, 4HNE, 4HNE plus midi-GAGR, or SU5402 plus midi-GAGR.

For cell viability/cytotoxicity measurement, neurons on glass coverslips were incubated in 1 x PBS containing 2 μM calcein AM (live cells: green) and 4 μM ethidium homodimer-1 (dead cells: red) for 10 min at 37°C. Immediately thereafter, neurons were imaged by 10 x objective using a fluorescence Olympus IX71 microscope (Olympus America Inc., Center Valley, PA) and, for the acquisition of high-quality images, using TCS SP5 multi-photon laser scanning confocal microscope.

### Co-culturing of microglial cells and primary rat cortical neurons

Microglial cell culture was prepared from the whole brain tissues (except of the cerebellum) of rat pups at postnatal day 3 (P3). Briefly, whole brain tissues except of the cerebellum were dissected and re-suspended in L-15 media on ice. Brain tissues were centrifuged at 1,000 x *g* for 3 min at 4°C. After the supernatant over brain tissue pellet was removed, the pellet was re-suspended in fresh L-15 media, followed by mechanical digestion using pasteur pipette. After the digestion, the resuspension was filtered through cell strainer (pore diameter = 70 μm). The flow-through was centrifuged at 1,000 x *g* for 3 min at 4°C. Then, cell pellet was re-suspended in DMEM media containing 10% FBS and 1X penicillin/streptomycin and plated at a high density in T75 culture flasks. The medium was exchanged with fresh medium every four days. After 8–10 days, culture flask caps were covered with parafilm to prevent gas exchange with environmental air. Flasks were then shaken in an orbital shaker at 220 rpm for 4 h at 37°C. Media was then collected into a conical tube and centrifuged at 800 x *g* for 10 min. The resulting cell pellet (mostly microglial cells) was then re-suspended in a neurobasal media containing B27 supplement and plated in filter insert (0.4-μm pore diameter) at a density of 2 x 10^5^ cells. The filter inserts containing microglial cells were transferred to a 24 well plate containing rat cortical neuron cultures (DIV6) at the bottom of each well. Microglial cells were treated with either 2 μM Aβ_42_ peptide or vehicle (H_2_O) and neurons with either 1 μM midi-GAGR or vehicle (H_2_O) for 48 h prior to live-dead assay. In addition, we examined whether microglial cells penetrated the filter and fell down to neurons at the bottom of well or not by staining the cells on coverslips at the bottom of well by staining the coverslips with anti-Iba1 antibody for immunocytochemistry and confocal microscopy.

### Analysis of neurite outgrowth and pCREB in primary mouse cortical neurons

To analyze the effect of polysaccharides on neurite outgrowth, primary mouse cortical neurons were treated with vehicle (H_2_O) or 1 μM of dextran, alginate, or midi-GAGR, 0.01 μM of HA-GAGR, or 0.1 μM of LA-GAGR for 2 days prior to immunocytochemistry using anti-α-tubulin antibody and secondary Alexa_488nm_ antibody. An etched grid coverslip containing 200 numbered boxes was used to select neurons objectively for image acquisition and analysis. A total of 24 boxes were randomly selected per treatment group. All the neurons having total neurite length longer than 4 times of the diameter of neuron cell body were chosen for image analysis. The total length of the neurites of each neuron was measured using Metamorph and used to calculate average total neurite length. To examine the effect of polysaccharides on the phosphorylation (activation) of nuclear CREB, neurons were stained with anti-pCREB antibody (Abcam & Santa Cruz biotechnologies, 2nd antibody with Alexa_568nm_), anti-α-tubulin antibody (Sigma, 2nd antibody with Alexa_488nm_), and DAPI (Sigma) after two-day incubation with vehicle (H_2_O) or 1 μM of dextran, alginate, or midi-GAGR, 0.01 μM of HA-GAGR, or 0.1 μM of LA-GAGR. Then, to identify midi-GAGR-induced signaling pathway that induces CREB phosphorylation, primary mouse cortical neurons were pre-treated with the inhibitors of FGFR1 (SU5402 [Santa Cruz Biotechnologies], 4 μM), PKC (staurosporine [Sigma], 3 nM), MEK (U0126 [Sigma], 10 μM), PI3K (LY294002 [Sigma], 20 μM), CaMKII (KN-62 [Calbiochem, Billerica, MA], 10 μM), or FAK (PF-573228 [Sigma], 1 μM) for 6 h and then with 1 μM midi-GAGR for 48 h prior to the staining of pCREB and α-tubulin. The images of neurons on coverslips were taken by confocal microscopy at the same gain (850), offset (-0.01) and exposure time (2 sec). The intensity of the staining of nuclear pCREB was measured using Metamorph and used to calculate average intensity. In addition, we detected the phosphorylation of CREB in the cytosols of mouse cortical neurons treated with polysaccharides by immunoblotting. Primary mouse neurons were dissected from 16 mouse embryos (E17) and plated in the wells of 6-well plates (1 x 10^6^ cells/well), differentiated for 6 days, and treated with polysaccharides for 48 h. Then, neurons were harvested and lysed in 1% Igepal CA-630 (Sigma)-containing PMEE buffer plus protease and phosphatase inhibitor cocktails (Sigma) for protein extraction. Extracted proteins were separated in 4–12% NuPAGE Bis-Tris protein gels (Life Technologies) and transferred to nitrocellulose membrane (GE Healthcare Life Science, Pittsburg, PA) using a semidry blotter (Hoefer, Inc. San Francisco, CA). The protein bands on blots recognized by anti-pCREB (Santa Cruz Biotechnologies) and anti-CREB antibodies were detected on Amersham Hyperfilm^™^ ECL films (GE Healthcare Life Science) using SuperSignal^®^ West Pico Chemiluminescent Substrate (Thermo Scientific).

### Examination of the in vivo neurotrophic effect of midi-GAGR

40 μL of 1 mM midi-GAGR or sterile H_2_O (vehicle) was administered intranasally into the nostrils (20 μL/nostril) of SD rats (4 rats per each) using a pipette. Animals were kept in anesthetized condition using 4% isoflurane and at supine position during administration to prevent the squirting-out of drug. Animals were sacrificed by decapitation at 6 h, 24 h, or 48 h after the administration. Whole brain was micro-dissected into the frontal cortex, hippocampus, and the rest of brain. Then, tissues were homogenized in the 2-fold volume of 1 x PMEE buffer containing 1% Igepal CA-630 and protease inhibitor cocktail using a 2-mL Teflon homogenizer. The homogenization was then incubated on ice for 30 min at 4°C, followed by centrifugation at 14,500 x *g* for 30 min. The supernatant was collected and its protein concentration was measured by Bradford assay. 20 μg of proteins was loaded onto each well of NuPage 4–11% Bis-Tris protein gels. Immunoblotting was performed using the antibodies to NF200, GAP-43, and GAPDH. The densities of protein bands were measured using Image J and normalized to that of the loading control, GAPDH. Normalized values were used to calculate average normalized band densities.

### Examination of the interaction of midi-GAGR with FGFR1 in brain synaptosomal plasma membrane

To examine whether midi-GAGR interacts with synaptosomal FGFR1 or not, we performed affinity chromatography using midi-GAGR-conjugated sepharose beads. We conjugated either midi-GAGR or dextran to epoxy-activated sepharose 6B that is a pre-activated medium that can be conjugated to the hydroxyl groups of carbohydrates. Briefly, 200 μL of 7.4 mM midi-GAGR or 5kD dextran in H_2_O was mixed with 200 μL of epoxy-activated sepharose beads in a microtube and incubated on a rotator (16 h, 37°C). The mixture was spun down at 1,000 x *g* (10 min) to separate bead-bound polysaccharides from unbound. Unoccupied active sites on beads were blocked by incubation (4 h, 45°C) in 1 M ethanolamine (pH 8). Then, beads were washed with three cycles of alternating pH solutions– 0.1 M acetate buffer (pH 4) and 0.1 M Tris-HCl buffer (pH 8), both containing 0.5 M NaCl. The conjugation of polysaccharides to beads was confirmed by phenol—sulfuric acid colorimetry [87]. Cerebral cortices were dissected from four female mice (BALB/C, 8 wks old) and homogenized with a hand grinder in 1 mL of PMEE homogenization buffer plus 1% Igepal CA-630 and protease inhibitors (PIs). The homogenate was centrifuged at 1,000 x *g* (10 min) to remove nuclei and undisrupted cells. The supernatant was subjected to 5–6 strokes through 27G needle. Post-nuclear supernatant was centrifuged at 1,000 x *g* (15 min). The supernatant was diluted to 1:2 with Igepal CA-630-free PMEE buffer (to make 0.5% Igepal CA-630) and stored at -80°C until use. Then, the supernatant was incubated with 100 μL of either midi-GAGR-conjugated or dextran-conjugated beads. After 24-h incubation on a rotator at 4°C, the beads were washed three times with 0.5 mL of PMEE buffer to remove proteins that nonspecifically bind to beads. Then, the beads were boiled for protein elution in a SDS loading buffer. Eluted proteins were separated in a SDS-NuPAGE gel and processed for immunoblotting using FGFR1 antibody.

### Examination of the effects of midi-GAGR on neuronal activity and neurodegenerative markers in 3xTg AD mice

We purchased 12-week-old 3xTg-AD mice (female, ~20 g, B6.Cg-Psen1tm1Mpm Tg [APPSwe, tauP301L]1Lfa/J) to examine the efficacy of midi-GAGR in restoring neuronal activity and reducing neurodegeneration in AD mouse brains. Three 3xTg AD mice were intranasally administered with either sterile H_2_O (vehicle) or 1 mM midi-GAGR (40 μL total, 20 μL/nostril) every day for 14 days after 4% isoflurane anesthetization and then sacrificed by decapitation. Whole brain was micro-dissected to obtain the cortices and hippocampi. The tissues were homogenized in the 2-fold volume of 1 x PMEE buffer containing 1% Igepal CA-630 plus phosphatase and protease inhibitor cocktail using a miniature cell grinder for 1.5 mL microtube. The homogenization was then incubated on a rotator for 30 min at 4°C, followed by centrifugation at 14,500 x *g* for 30 min. The supernatant was collected and its protein concentration was measured by Bradford assay. 30 μg of proteins was loaded onto NuPage 4–11% Bis-Tris protein gels. Immunoblotting was performed using the antibodies to NF200, GAP-43, PSD95, synaptophysin, pCREB, CREB, p-tau (AT8), tau, and GAPDH. The densities of protein bands were measured using Image J and normalized to that of the loading control, GAPDH. Normalized values were used to calculate average normalized band densities.

### Statistical Analysis

All cell culture experiments were replicated multiple times with different batches of cell cultures. Microscopic analysis was performed blindly by students. Statistical significance between two groups was calculated using unpaired student’s *t*-test with a value of p<0.05 that was considered statistically significant. Multiple comparisons were performed using one-way ANOVA followed by Dunnett’s or Bonferroni’s multiple comparisons tests (GraphPad Prism software, La Jolla, CA).

## Results

### Generation of small-size polysaccharides from low acyl gellan gum

We had searched for a neuroprotective polysaccharide among those that are currently used in human as food additive. Then, we found the strong neuroprotective polysaccharide, low acyl gellan gum (LA-GAGR). 1 μM LA-GAGR protected differentiated N2A cells and their neurites from high doses of 4HNE (data not shown), a reactive lipid radical that causes neurodegeneration [88, 89]. In order to increase water solubility and diffuse-ability, we cleaved LA-GAGR into smaller sizes by enzymatic digestion (α(1→3)-glycosidase) for 24, 48, and 72 h. The MWs of its cleavage products were determined using Parallel Plate Rheometer that measures shear storage modulus and loss modulus and yields the viscosity profile of polysaccharides. From the viscosity-storage modulus profiles, the MW distributions of the cleavage products were determined using RheoAnalyzer program. The validity of the RheoAnalyzer program was verified by running polystyrene standard (NBS 706) on the Parallel Plate Rheometer and determining MW from the viscosity profile. The MW distributions of LA-GAGR and its 24-h, 48-h, and 72-h digestions are shown in ‘S1 and S2 Appendices’. The average MW of LA-GAGR was ~99,639 g/mole that is close to the value reported by CPKelco Co. The MWs of 24-h, 48-h, and 72-h cleavage products were ~30,245 g/mole, ~4,775 g/mole, and ~718 g/mole, respectively. The average MW of ~4,775 g/mole (named ‘midi-GAGR’) is equivalent to six repeating units and that of ~718 g/mole (named ‘mini-GAGR’) is to one repeating unit. Two small-size LA-GAGR products, midi-GAGR and mini-GAGR, were chosen for further examination regarding their neuroprotective effect.

### Midi-GAGR rescues neurites from the atrophy caused by 4HNE and H_2_O_2_

**Fig 2:**
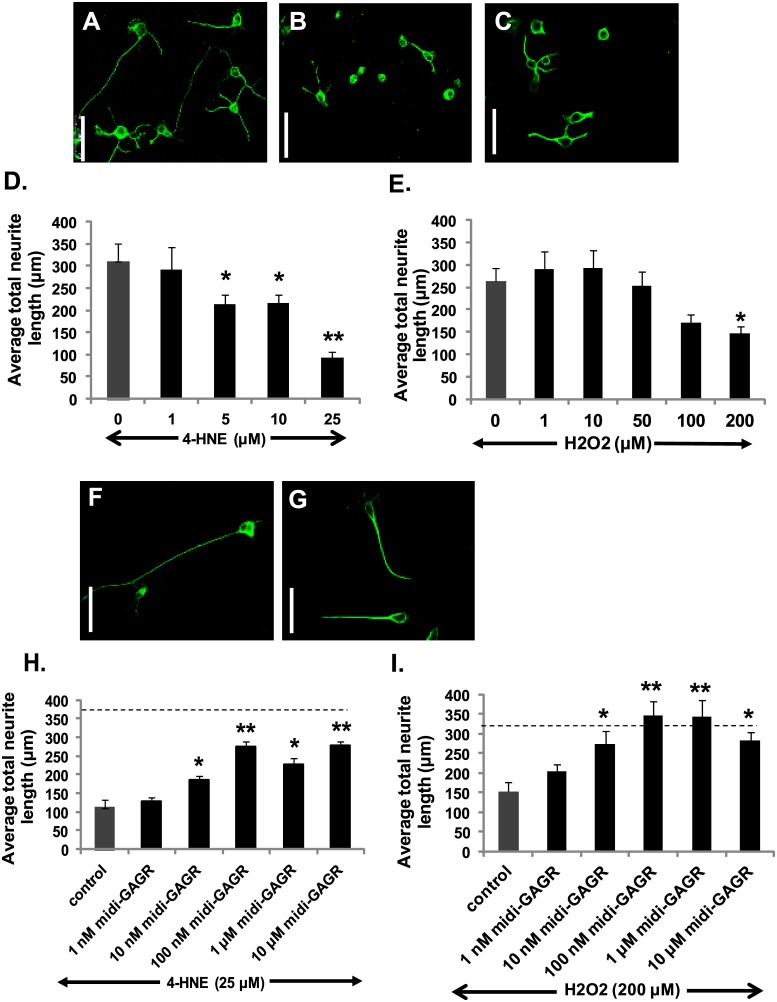
Midi-GAGR reverses neurite atrophy caused by 4HNE and H_2_O_2_. (A-E) Differentiated N2A cells were treated with different concentrations of 4HNE (0, 1, 5, 10 and 25 μM) for 48 h or H_2_O_2_ (0, 1, 10, 50, 100 and 200 μM) for 24 h and immunostained with α-tubulin antibody. The representative images of N2A cells treated with H_2_O (vehicle) (A), 25 μM 4HNE (B), or 200 μM H_2_O_2_ (C). Scale bar = 75 μm. Bar graphs represent the average total neurite lengths of N2A cells in response to different concentrations of either 4HNE (D) or H_2_O_2_ (E). *, p < 0.05 and **, p < 0.001 compared to control. (F-I) Differentiated N2A cells were pre-treated with different concentrations (0, 0.001, 0.01, 0.1, 1, and 10 μM) of midi-GAGR for 24 h, followed by incubation with either 25 μM 4HNE for 48 h or 200 μM H_2_O_2_ for 24 h and then immunostained with anti-α-tubulin antibody. The representative images of N2A cells pretreated with 1 μM midi-GAGR and then incubated with either 25 μM 4HNE (F) or 200 μM H_2_O_2_ (G). Scale bar = 75 μm. Bar graphs show the average total neurite lengths of N2A cells pre-treated with different concentrations of midi-GAGR followed by treatment with 25 μM 4HNE (H) or 200 μM H_2_O_2_ (I). Dotted lines correspond to the average total neurite lengths of N2A cells without any treatment. Data represent mean ± SEM of, at least, 40 cells/group from two independent experiments. *, p<0.05 and **, p<0.001 compared to 4HNE alone.

We used differentiated N2A cells to examine if midi-GAGR and mini-GAGR protect neurites from the oxidative insults of 4HNE and H_2_O_2_ as LA-GAGR does. We, first, determined the atrophic dose ranges of the radicals by treating differentiated N2A cells with increasing concentrations of 4HNE and H_2_O_2_ for 48 h and 24 h, respectively. Treated cells were fixed, immunostained by anti-α-tubulin antibody, imaged by confocal microscopy, and examined regarding total neurite length. The average total neurite length of N2A cells was decreased in a dose-dependent manner in response to 4HNE and H_2_O_2_ to maximum extents at 25 μM 4HNE (Fig 2B and 2D) and 200 μM H_2_O_2_ (Fig 2C and 2E) compared to vehicle (Fig 2A). It suggests that 25 μM and 200 μM are the maximum doses of 4HNE and H_2_O_2_, respectively, that causes almost complete neurite atrophy in differentiated N2A cells. Then, we treated differentiated N2A cells with increasing concentrations of midi-GAGR prior to the treatment with either 25 μM 4HNE or 200 μM H_2_O_2_. Treatment with 0.1 and 1 μM midi-GAGR prior to 4HNE treatment rescued neurites up to ~70% of the control level (vehicle) (Fig 2F and 2H). Similarly, treatment with 0.1 and 1 μM midi-GAGR prior to H_2_O_2_ treatment rescued neurites up to ~100% of the control level (Fig 2G and 2I). We also examined the protective effect of mini-GAGR but found that mini-GAGR was not as potent as midi-GAGR in protecting neurites from the free radicals (data not shown). Therefore, we chose midi-GAGR that showed stronger neuroprotective effect against 4HNE and H_2_O_2_ for further study.

### Midi-GAGR reduces the apoptosis of rodent cortical neurons caused by 4HNE, H_2_O_2_, and amyloid β peptide

**Fig 3:**
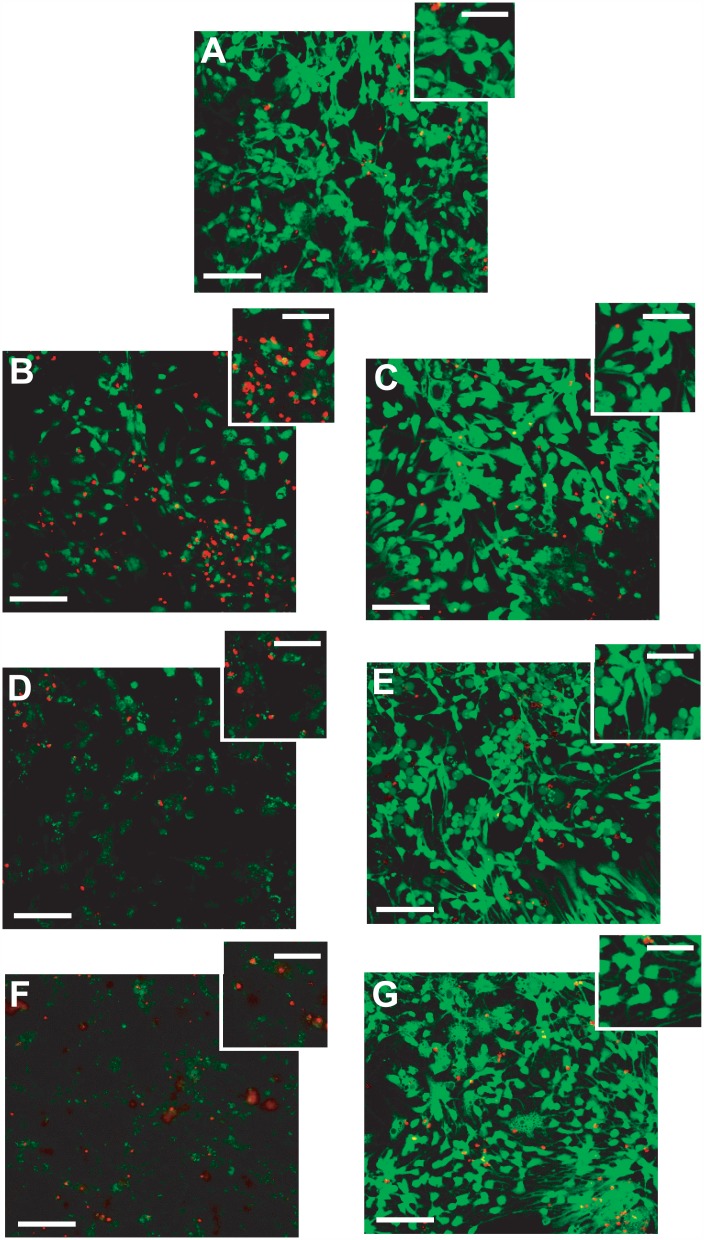
Pre-treatment with midi-GAGR significantly reduces the apoptosis of rodent cortical neurons caused by 4HNE, H_2_O_2_, and amyloid β peptide. (A-G) Mouse cortical neurons at DIV5 were pre-treated with H_2_O (vehicle), 1 μM midi-GAGR, 0.1 μM LA-GAGR, 0.01 μM HA-GAGR, 1 μM alginate or 1 μM dextran followed by incubation with vehicle, 10 μM 4HNE, or 50 μM H_2_O_2_ for 24 h or 2 μM Aβ_42_ for 48 h. After the treatment, neurons were processed for live/dead assay using calcein AM and ethidium homodimer-I. The representative images of H_2_O (A), 4HNE alone (B), 4HNE + midi-GAGR (C), H_2_O_2_ alone (D), H_2_O_2_ + midi-GAGR (E), Aβ_42_ alone (F), and Aβ_42_ + midi-GAGR (G). Live cells were labeled as green and dead cells as red. Scale bar = 100 μm. Insets show the magnified images of individual neurons. Inset scale bar = 50 μm.

**Fig 4:**
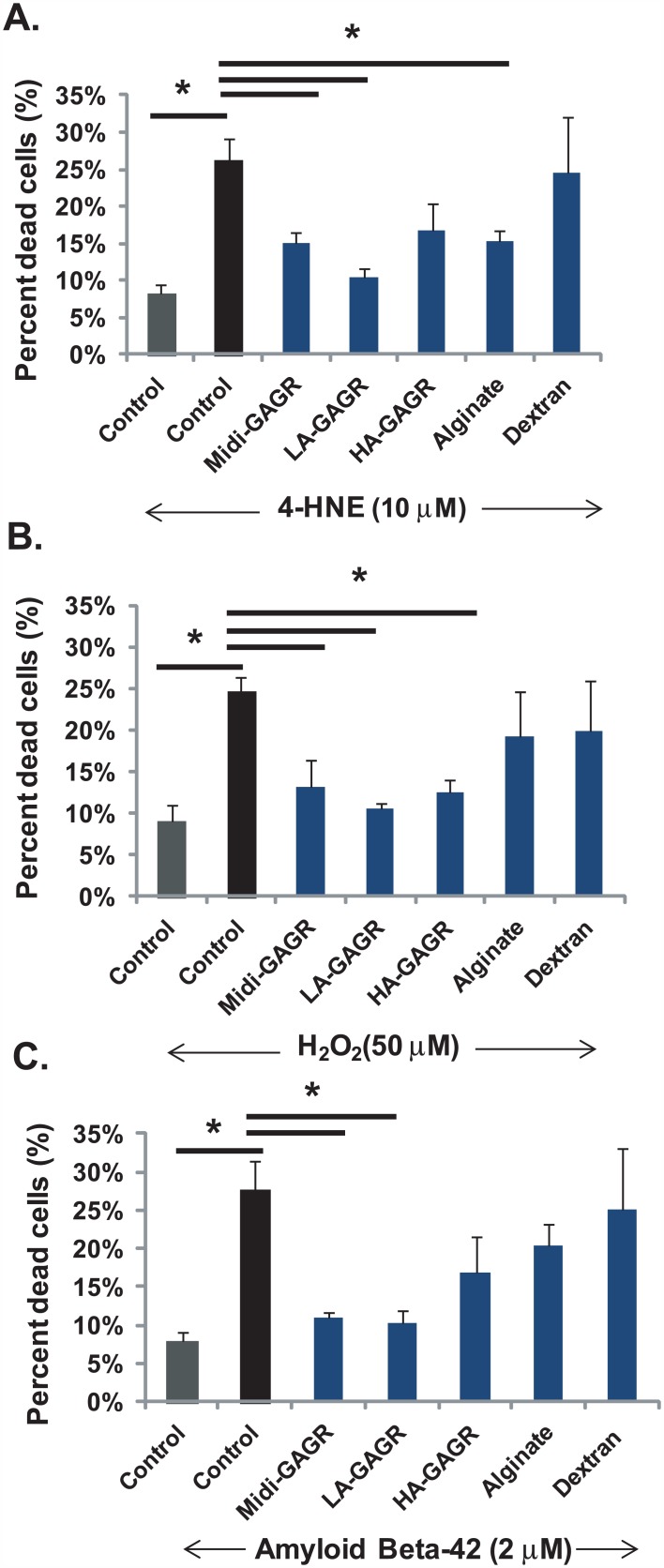
Quantification of neuron death in response to oxidative insults in the presence of different polysaccharides. The numbers of live and dead cells were counted using Metamorph software. Bar graphs represent the percents of dead neurons after the pre-treatments with different polysaccharides followed by treatment with 4HNE (A), H_2_O_2_ (B), or Aβ_42_ (C). Data represent mean ± SEM of three independent experiments. For each experiment, at least, 1,000 cells per group were counted. *, p<0.05.

Based on our observation that midi-GAGR attenuated neurite atrophy caused by free reactive radicals, we speculated that midi-GAGR might protect neurons from the death caused by free radical insults. Thus, we examined the extent to which midi-GAGR protects the primary culture of rodent cortical neurons from 4HNE and H_2_O_2_. In addition to the radicals, we tested amyloid β peptide (Aβ_42_) because Aβ_42_ peptide is a major causative factor that causes oxidative stress and neuron death [90, 91]. We treated mouse cortical neurons (E17, DIV5) with 10 μM 4HNE (24 h), 50 μM H_2_O_2_ (24 h), or 2 μM Aβ_42_ peptide (48 h) after the treatment of the neurons with vehicle (H_2_O), midi-GAGR (1 μM), dextran (1 μM), alginate (1 μM), LA-GAGR (0.1 μM), or HA-GAGR (0.01 μM) for 24 h. The concentrations of 4HNE, H_2_O_2_, and Aβ_42_ peptide were chosen according to their patho-physiological concentrations [89, 92–95]. The concentrations of LA-GAGR and HA-GAGR were chosen because, within the same volume, the total numbers of sugar units in the polysaccharides at the concentrations are close to that of 1 μM midi-GAGR. The viability of neurons was assessed using LIVE/DEAD^®^ Viability/Cytotoxicity Assay Kit in which membrane-permeant calcein AM is cleaved by esterase in live cells, thus yielding green fluorescence, and membrane-impermeant ethidium homodimer-1 stains the nucleic acids of plasma membrane-compromised cells with red fluorescence. We counted the numbers of green (live) and red (dead) neurons in each condition using Metamorph. Although the intensities of green fluorescent signals in neurons treated with either free radicals or Aβ_42_ peptide were weak, we included the neurons in our counting. About 8–9% of vehicle-treated neurons died during the process of live/dead cell assay (Figs 3A and 4). Upon exposure to 10 μM 4HNE, ~26% of mouse cortical neurons died (Figs 3B and 4A) while pre-treatment with 1 μM midi-GAGR (Fig 3C) and 0.1 μM LA-GAGR reduced neuron death to 14% and 10%, respectively (Fig 4A). HA-GAGR and alginate also reduced the percent of neuron death caused by 4HNE while dextran did not (Fig 4A). Exposure to 50 μM H_2_O_2_ caused neuron death in ~25% of cortical neurons that were pre-treated with vehicle (Figs 3D and 4B). Pre-treatment with either alginate or dextran did not reduce H_2_O_2_-caused neuron death (Fig 4B). Conversely, pre-treatment with midi-GAGR (Fig 3E), LA-GAGR, or HA-GAGR reduced neuronal death to ~13% (Fig 4B). These results suggest that midi-GAGR, LA-GAGR, and HA-GAGR can protect rodent cortical neurons from both H_2_O_2_ and 4HNE while dextran and alginate cannot.

We, then, examined the extent to which midi-GAGR protects cortical neurons from Aβ_42_ peptide. Exposure to 2 μM Aβ_42_ peptide caused the death of about 30% of neurons pre-treated with vehicle (Figs 3F and 4C). Pre-treatment with HA-GAGR, alginate, or dextran did not decrease the percent of neuron death compared to pre-treatment with vehicle (Fig 4C). Conversely, pre-treatment with either midi-GAGR or LA-GAGR reduced neuron death to ~10%, the control level (Figs 3G and 4C). Thus, only midi-GAGR and LA-GAGR can protect cortical neurons from Ab_42_ peptide while other polysaccharides cannot.

### Midi-GAGR reduces the apoptosis of rodent cortical neurons from co-treated 4HNE or Aβ_42_ peptide

**Fig 5:**
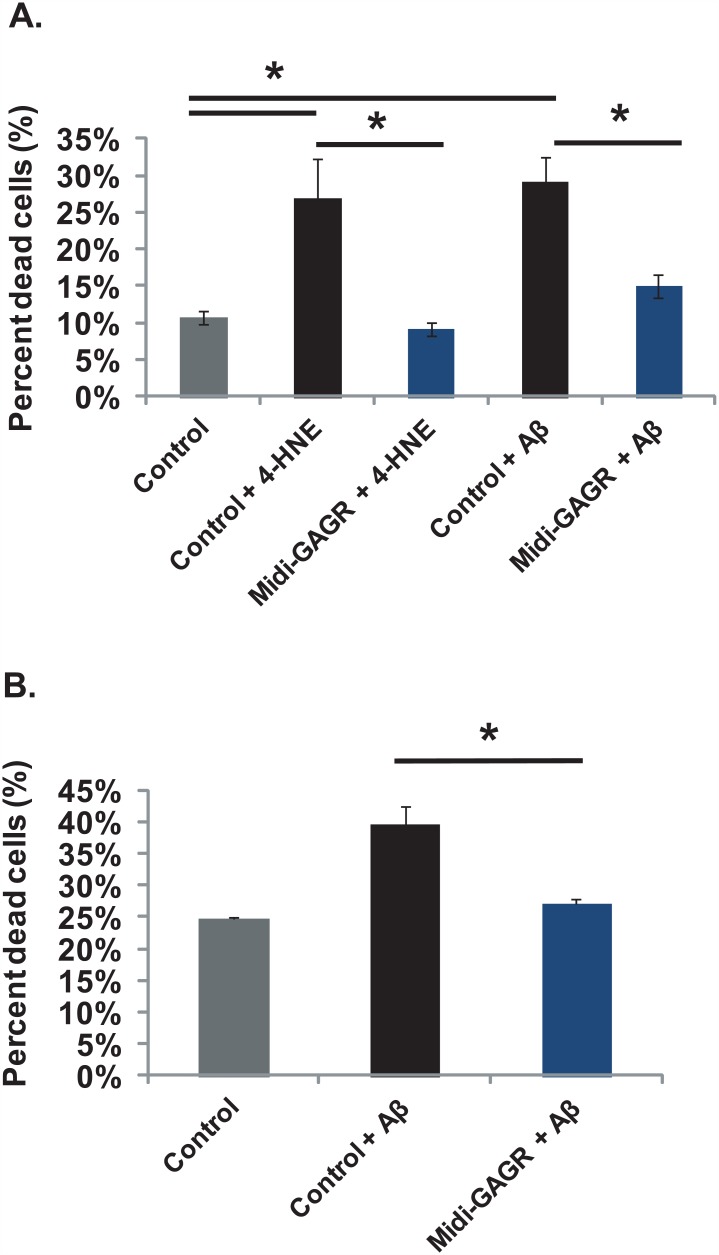
Midi-GAGR protects rodent cortical neurons from co-treated 4HNE, Aβ_42_ peptide and activated microglial cells. (A) Rat cortical neurons (E17) at DIV5 were co-treated with either 10 μM 4HNE (for 24 h) or 2 μM Aβ_42_ (for 48 h) and either water or 1 μM midi-GAGR. After the treatment, neurons were processed for live/dead assay using calcein AM and ethidium homodimer-I. Live and dead cells were imaged using a fluorescence microscope. The numbers of live and dead cells were counted using Metamorph software. Bar graphs show the percents of dead neurons after co-treatment with either 4HNE or Aβ_42_ plus/minus midi-GAGR. Data represent mean ± SEM of three independent experiments. *, p<0.05. (B) The co-cultures of rat cortical neurons and microglia cells were treated with 2 μM Aβ_42_ plus/minus 1 μM midi-GAGR. After 48 h, transwell filters containing microglial cells were removed and neurons in bottom wells were processed for live/dead assay. Live and dead cells were imaged using a fluorescence microscope. The numbers of live and dead neurons were counted using Metamorph software. Bar graphs show the percent of dead neurons. Data represent mean ± SEM of three independent experiments. *, p<0.05.

It is likely that free reactive radicals already exist at pathological concentrations inside the brain before some symptoms are noticeable for treatment. In other words, treatment is likely to start at the pre-existence of the pathological concentrations of the radicals. Thus, we examined if midi-GAGR can protect cortical neurons from co-treated 4HNE or Aβ_42_ peptide. Rat cortical neurons (E17, DIV5) were treated with either 10 μM 4HNE (24 h) or 2 μM Aβ_42_ peptide (48 h) and either vehicle or 1 μM midi-GAGR. Then, the viability of neurons was assessed using LIVE/DEAD^®^ Viability/Cytotoxicity Assay Kit. Exposure to 10 μM 4HNE caused apoptosis in ~27% of vehicle-treated neurons while co-treatment with midi-GAGR reduced the percent of neuron death to ~9% (Fig 5A). Treatment with 2 μM Aβ_42_ peptide caused death in ~29% of vehicle-treated neurons while co-treatment with midi-GAGR reduced the percent of neuron death to ~ 15% (Fig 5A). These results suggest that midi-GAGR can also protect rodent cortical neurons from co-treated 4HNE or Aβ_42_ peptide.

### Midi-GAGR protects rodent cortical neurons from microglial cells activated by Aβ_42_ peptide

Microglial cells activated by Aβ_42_ peptide are a major causative factor for neurodegeneration, especially AD [96]. As such, activated microglial cells secrete free radicals and pro-inflammatory cytokines that facilitate neurodegeneration and neuron apoptosis. Thus, we examined if midi-GAGR protects primary rodent cortical neurons from microglia cells activated by Aβ_42_ peptide. Microglial cells were isolated from rats on the postnatal day 1 (S3 Appendix) and seeded in 0.4μm-pore-size filter insert that fits into the well of 24-well plate. The filter inserts were transferred to a 24-well plate in which primary rat cortical neurons (E17) were cultured at the bottoms of wells for 6 days (DIV6). Then, microglia cells were treated with 2 μM Aβ_42_ and neurons with either vehicle or 1 μM midi-GAGR. After 48 h, the viability of neurons was assessed using LIVE/DEAD Viability/Cytotoxicity Assay Kit. Around 25% of the neurons treated with vehicle died under untreated microglial cells (Fig 5B). Upon treatment of microglial cells with Aβ_42_ peptide, the percent of death in neurons treated with vehicle was increased to ~40%. Conversely, treatment of neurons with 1 μM midi-GAGR reduced death to ~27% that was close to the percent of dead neurons under untreated microglial cells (Fig 5B). Thus, it is clear that midi-GAGR can protect rodent cortical neurons from activated microglial cells.

### Midi-GAGR enhances neurite outgrowth in N2A cells and rodent cortical neurons

**Fig 6:**
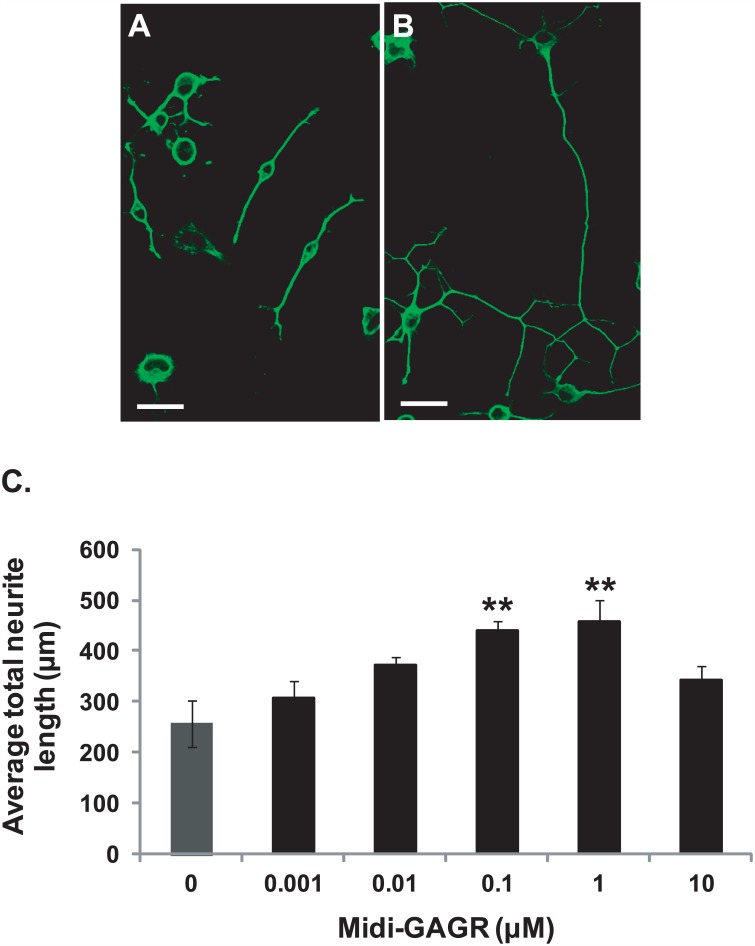
Midi-GAGR enhances neurite outgrowth in N2A cells. Differentiated N2A cells were treated with different concentrations of midi-GAGR for 48 h and immunostained using anti-α-tubulin antibody. Representative confocal images of N2A cells treated with either H_2_O (vehicle) (A) or 1 μM midi-GAGR (B). Scale bar = 30 μm. (C) Bar graphs show the average total neurite lengths of N2A cells treated with different concentrations of midi-GAGR (mean ± SEM of, at least, three independent experiments). *, p<0.05 compared to control.

**Fig 7:**
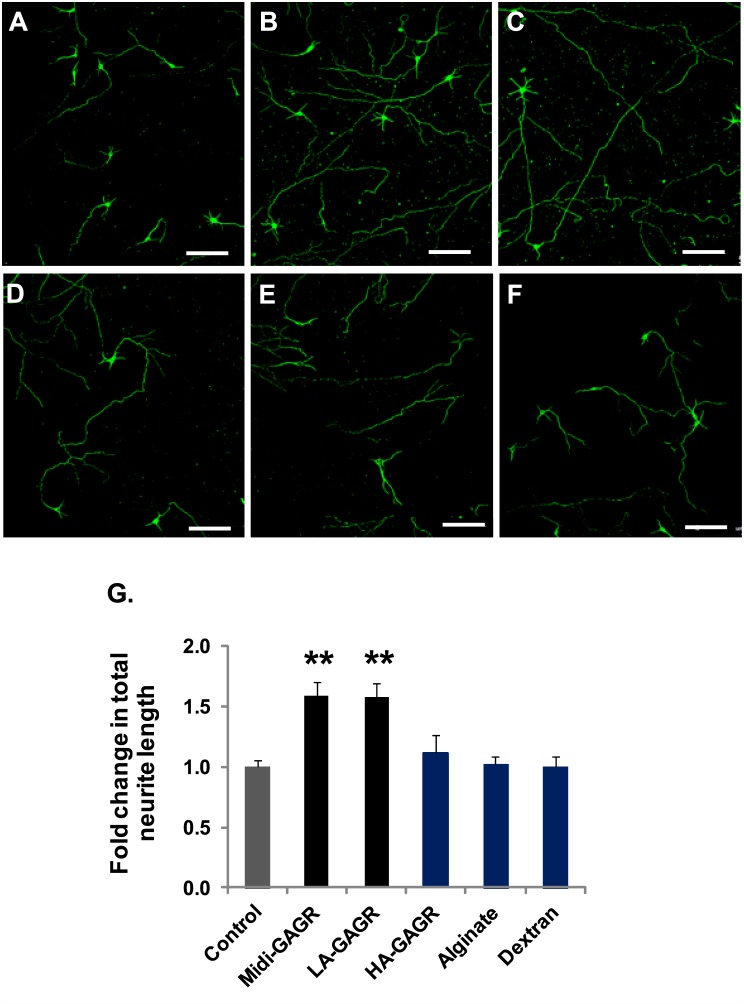
Midi-GAGR enhances neuritogenesis in mouse cortical neurons. (A-F) Mouse cortical neurons (E17, DIV4) were treated with H_2_O (vehicle) (A), 1 μM midi-GAGR (B), 0.1 μM LA-GAGR (C), 0.01 μM HA-GAGR (D), 1 μM alginate (C), or 1 μM dextran (F) for 48 h and immunostained with anti-α-tubulin antibody. Scale bar = 100 μm. (G) Bar graphs show average fold changes in the total neurite length of mouse cortical neurons in response to different polysaccharides (mean ± SEM of, at least, three independent experiments). **, p<0.01 compared to control.

In addition to the neuroprotective effect, we found that LA-GAGR enhanced neurite outgrowth in N2A cells. Thus, we expected that midi-GAGR would have a similar enhancing effect on neuritogenesis. First, we examined the neuritogenic effect of midi-GAGR on N2A cells. N2A cells were differentiated with increasing concentrations (0, 0.001, 0.01, 0.1, 1, and 10 μM) of midi-GAGR for 48 h. Then, cells were fixed and immunostained with anti-α-tubulin antibody. The total length of neurites per cell was measured using Metamorph and used to calculate average total neurite length per treatment group. At 0.1 and 1 μM, the average total neurite length of midi-GAGR-treated N2A cells reached ~1.7 fold higher than that of vehicle-treated cells (Fig 6; 438.21±20.55 μm [0.1 μM] and 457.76±41.66 μm [1 μM] vs. 257.51±45.16 μm [vehicle], p<0.05). At 10 μM, average total neurite length was decreased, which is similar to the pattern of neuritogenesis in cells treated with FGL [79]. We also examined the neuritogenic effect of midi-GAGR on primary mouse cortical neurons (E17, DIV4). Neurons were incubated with vehicle, midi-GAGR (1 μM), dextran (1 μM), alginate (1 μM), LA-GAGR (0.1 μM), or HA-GAGR (0.01 μM) for 2 days and immunostained with the antibody to βIII tubulin (Fig 7A–7F). The total neurite length of each neuron was measured to calculate average total neurite length per condition. Then, average total neurite length per condition was divided by that of vehicle treatment to obtain fold change in average total neurite length per condition. Compared to vehicle treatment (Fig 7A), midi-GAGR (Fig 7B) and LA-GAGR (Fig 7C) increased average total neurite length by ~1.6 fold (Fig 7G). Conversely, HA-GAGR (Fig 7D), alginate (Fig 7E), and dextran (Fig 7F) did not enhance neuritogenesis (Fig 7G). Thus, only midi-GAGR and LA-GAGR have strong neuritogenic effect on rodent cortical neurons.

### Midi-GAGR activates CREB, a neurotrophic transcriptional factor

**Fig 8:**
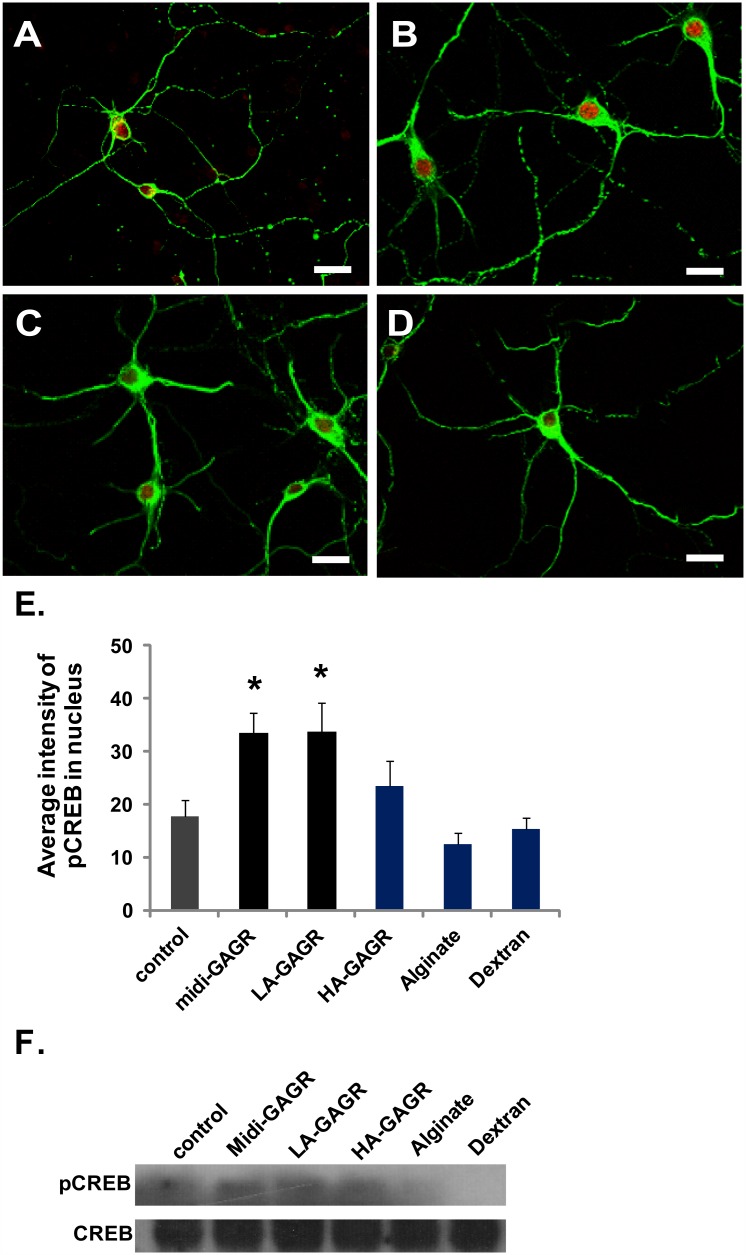
Midi-GAGR activates CREB, a neurotrophic transcriptional factor. Mouse cortical neurons (DIV4) were treated with H_2_O (vehicle), 1 μM midi-GAGR, 0.1 μM LA-GAGR, 0.01 μM HA GAGR, 1 μM alginate, or 1 μM dextran for 48 h and immunostained with DAPI (not shown) and the antibodies to α-tubulin and pCREB. The representative images of neurons treated with H_2_O (A), midi-GAGR (B), alginate (C) or dextran (D), followed by staining with α-tubulin (green) and pCREB (red). Scale bar = 30 μm. (C) Bar graphs show the average intensities of pCREB after different treatments (n = 90 neurons, mean ± SEM of, at least, three independent experiments). *, p<0.05 compared to control. (D) The cytosols extracted from neurons treated with H_2_O (vehicle), 1 μM midi-GAGR, 0.1 μM LA-GAGR, 0.01 μM HA GAGR, 1 μM alginate, or 1 μM dextran for 48 h were used for immunoblotting using the antibodies to pCREB and CREB (neurons extracted from sixteen E17 mouse embryos, n = 2 experiments).

Since both survival-enhancement and neuritogenesis converge on neurotrophic effect [33, 97], it is possible that midi-GAGR activates a neurotrophic signaling pathway that increases neuron survival in neurodegenerative condition and enhances neuritogenesis. To examine the possibility, we stained midi-GAGR-treated mouse cortical neurons with the antibody to pCREB, a marker for activated neurotrophic signaling pathways [98–100]. Mouse cortical neurons (E17, DIV4) were treated with vehicle (Fig 8A), midi-GAGR (1 μM, Fig 8B), alginate (1 μM, Fig 8C), dextran (1 μM, Fig 8D), LA-GAGR (0.1 μM), or HA-GAGR (0.01 μM) for 48 h, fixed, immunostained with the antibodies to α-tubulin and pCREB along with DAPI. The fluorescence intensity (arbitrary number) of stained pCREB in the nucleus was measured using Metamorph. Vehicle-treated neurons showed the basal levels of pCREB in the nuclei (Fig 8A). Conversely, treatment with either midi-GAGR (Fig 8B) or LA-GAGR significantly increased the level of nuclear pCREB while the other polysaccharides did not (Fig 8C and 8D). Treatment with HA-GAGR slightly increased the level of nuclear pCREB. We calculated the average intensity of stained pCREB in the nuclei of the neurons. Treatment with either midi-GAGR or LA-GAGR increased the average intensity of nuclear pCREB by ~2 fold compared to vehicle treatment (Fig 8E). To confirm the result of the optical measurement of the level of pCREB, we performed immunoblotting to detect the phosphorylation of CREB protein using the cytosols extracted from mouse cortical neurons treated with vehicle or polysaccharides. Total CREB was also detected by immunoblotting. Compared to control (vehicle treatment), the levels of pCREB were significantly increased in neurons treated with either midi-GAGR or LA-GAGR while those in neurons with either alginate or dextran were not (Fig 8F). The phosphorylation level of HA-GAGR was also increased. Thus, midi-GAGR and LA-GAGR increase the phosphorylation of CREB significantly and HA-GAGR does slightly.

### Intranasally administered midi-GAGR penetrates the BBB and increases the expression of NF200 and GAP-43 in the frontal cortex and hippocampus

**Fig 9:**
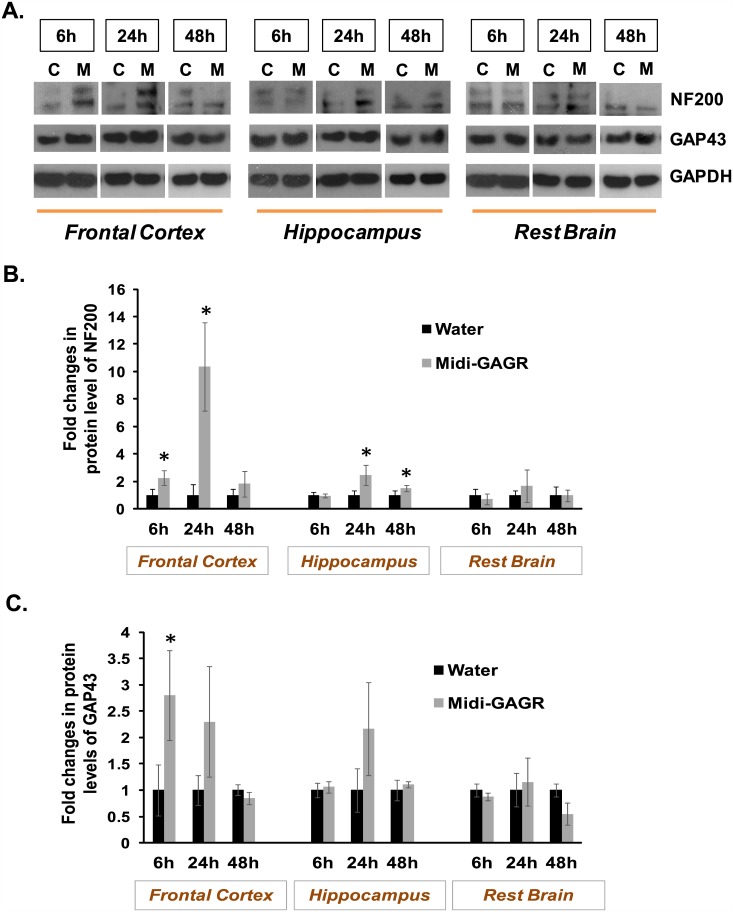
Intranasally administered midi-GAGR increases the expression of NF200 and GAP-43 in the brains of live rats. SD rats were intranasally administered with either vehicle or midi-GAGR and processed to obtain brains at 6h, 24h or 48h after the administration. Brains were dissected to the frontal cortex, hippocampus, and rest of the brain. (A) Brain tissue lysates were processed for immunoblotting using the antibody to NF200 (upper panel), GAP-43 (middle panel), or GAPDH (lower panel). ‘C’ is control and ‘M’ is midi-GAGR. The band densities of NF200 and GAP-43 were measured using image J software and normalized to those of GAPDH. Bar graphs show fold changes in the level of NF200 (B) and GAP-43 (C) in the different parts of brains at given time points. Data represents mean ± SEM (n = 4 animals/group). *, p<0.05.

Our previous study demonstrated that midi-GAGR penetrated the BBB and remained at 0.1–0.2 μM minside the brain for ~24 h after one-time intranasal administration [101]. Therefore, intranasally administered midi-GAGR is expected to exert a neurotrophic effect inside the brain within 24 h post-administration. We examined the expression of two neurotrophic protein markers, NF200 and GAP-43, in the brains of rats administered intranasally with 40 μL of either H_2_O or 1 mM midi-GAGR. At 6, 24, and 48 h after the intranasal administration, the frontal cortex, hippocampus, and the rest of the brain were dissected from the rats and processed for immunoblotting using the antibodies to NF200, GAP-43, and GAPDH (loading control). The protein band densities of NF200 and GAP-43 were measured by densitometry (Image J), normalized by those of GAPDH, and used to calculate average normalized values. In the frontal cortex, the expression level of NF200 was increased to the level significantly higher than control at 6 h and 24 h after the administration (Fig 9A and 9B). In the hippocampus, that of NF200 was increased to the level statistically higher than control after 24 h. The expression level of GAP-43 was also significantly increased in the frontal cortex at 6 h and 24 h while slightly increased in the hippocampus at 24 h (Fig 9A and 9C). The rest of the brain did not show an increase in NF200 and GAP-43 after one-time intranasal administration. These results indicate that intranasally administered midi-GAGR enters the brain and exerts its neurotrophic effect in the frontal cortex and hippocampus within 24 h post-administration.

### Midi-GAGR binds to FGFR1 and activates FGFR1 signaling pathway

**Fig 10:**
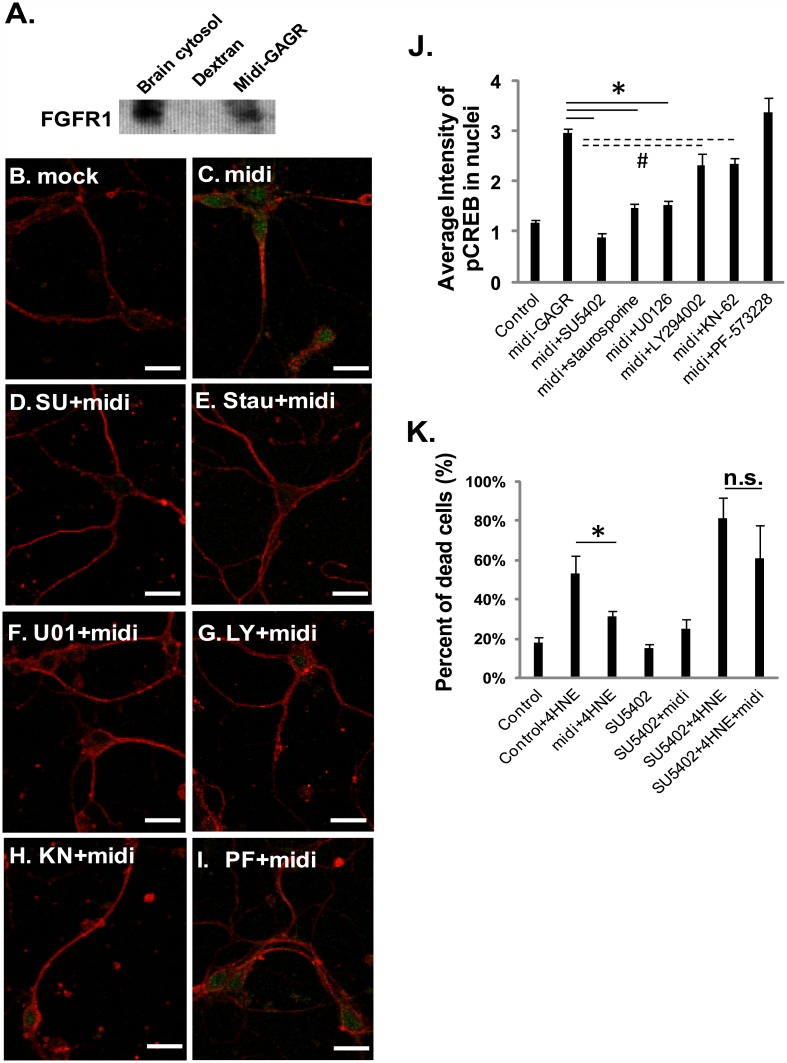
Midi-GAGR binds to FGFR1 and uses FGFR1 signaling pathway to activate CREB and protect neurons from the death caused by oxidative insult. (A) Midi-GAGR- or dextran-conjugated epoxy sepharose beads were mixed with synaptosomal plasma membrane proteins in 0.5% Igepal CA-630 PMEE buffer to pull down midi-GAGR-interacting FGFR1. Precipitated FGFR1 was detected by immunoblotting (n = 2, four rat brains). (B-J) Mouse cortical neurons (DIV4) were pre-treated with H_2_O (vehicle, B,C) or the inhibitors of FGFR1 (SU5402 [SU], 4 μM, D), PKC (staurosporine [Stau], 3 nM, E), MEK (U0126 [U01], 10 μM, F), PI3K (LY294002 [LY], 20 μM, G), CaMKII (KN-62 [KN], 10 μM, H), or PF-573228 (PF, 1 μM, I) for 6 h and then with mock (B) or 1 μM midi-GAGR (+midi, C-I) for 48 h. Neurons were then immunostained with the antibodies to α-tubulin (red) and p-CREB (green). Scale bar = 100 μm. (J) Bar graphs show the average intensities of pCREB after different treatments (n = 60 neurons, mean ± SEM). *, p<0.01 and #, p<0.05 compared to control. (K) Rat cortical neurons (E17, DIV6) were treated for 6 h with FGFR1 inhibitor (SU5402, 4 μM) and treated with 10 μM 4HNE and either vehicle or 1 μM midi-GAGR for 24 h prior to cell viability/cytotoxicity assay. As controls, neurons were treated with 4HNE, 4-HNE plus midi-GAGR, SU5402, midi-GAGR, or SU5402 plus midi-GAGR. Live and dead cells were imaged using a fluorescence microscope. The numbers of live and dead neurons were counted using Metamorph software. Bar graphs show the percent of dead neurons. Data represent mean ± SEM of three independent experiments. For each experiment, at least, 200 cells per group were counted. *, p<0.05 (n.s.: not significant).

Our pilot proteomics study hinted that midi-GAGR might interact with FGFR1 (data not shown). Thus, we examined the possible interaction of midi-GAGR with FGFR1 by affinity chromatography using midi-GAGR-conjugated sepharose column. Either midi-GAGR or dextran was conjugated to epoxy sepharose beads according to manufacturer’s protocol. Whole mouse brains were homogenized for cytosol extraction in PMEE buffer containing 1% Igepal CA-630 and protease inhibitor cocktail. Brain cytosols were diluted to 1:2 to make 0.5% Igepal buffer prior to the incubation with either dextran- or midi-GAGR-conjugated sepharose beads on a rotating plate for 16 h at 4°C. Nonspecific bindings were removed by extensive washes in PMEE buffer. Beads were boiled for protein elution. FGFR1 was one of the proteins eluted from midi-GAGR beads but not from dextran beads (Fig 10A).

As midi-GAGR binds to FGFR1, midi-GAGR is expected to activate FGFR1 signaling pathway, which results in CREB phosphorylation. We used pharmacological agents to inhibit signaling molecules downstream of FGFR1 that mediate CREB phosphorylation. We measured the fluorescence intensities of nuclear pCREB in mouse cortical neurons (E17, DIV6) pre-treated for 6 h with the inhibitors of FGFR1 (SU5402 [SU], 4 μM), PKC (staurosporine [Stau], 3 nM), MEK (U0126 [U01], 10 μM), PI3K (LY294002 [LY], 20 μM), or CaMKII (KN-62 [KN], 10 μM) and then with 1 μM midi-GAGR (midi) for 48 h. PF-573228 (PF, 1 μM) that inhibits FAK which sits at the bottleneck of the signaling pathway downstream of NCAM180 [73] was also included to examine whether NCAM180 is involved in midi-GAGR-mediated CREB phosphorylation or not. Treated neurons were stained with the antibodies to βIII tubulin (red) and pCREB (green) for the measurement of the average intensities of nuclear pCREB. Compared to neurons treated with vehicle (Fig 10B and 10J), those with midi-GAGR (midi) showed a significant increase (~2.6 fold) in the average intensity of pCREB (Fig 10C and 10J). Conversely, pre-treatment with the inhibitor of FGFR1 (Fig 10D and 10J), PKC (Fig 10E and 10J), or MEK (Fig 10F and 10J) significantly decreased the average intensity of pCREB (solid lines, *: p<0.01, n = 40–50 neurons). In neurons pre-treated with the inhibitor of either PI3K or CaMKII, the average intensity of pCREB was decreased slightly but still statistically significantly (dotted lines, #: p<0.05, n = 40–50 neurons). In contrast to the inhibitors, pre-treatment with FAK inhibitor did not decrease the average intensity of pCREB in midi-GAGR-treated neurons (Fig 10I and 10J). These results suggest that midi-GAGR activates FGFR1 and its downstream signaling pathways consisting of PKC, MEK, PI3K, and CaMKII, but not NCAM180-FAK signaling pathway for CREB phosphorylation.

Next, we examined how much FGFR1-mediated neurotrophic signaling pathway contributes to midi-GAGR-mediated neuroprotection against oxidative insult. We pre-treated rat cortical neurons (E17, DIV6) for 6 h with FGFR1 inhibitor (SU5402, 4 μM) and then with 10 μM 4HNE for 24 h prior to LIVE/DEAD Viability/Cytotoxicity Assay. Treatment with 4HNE increased the percent of dead cells from ~20% to ~60% while co-treatment with 1 μM midi-GAGR decreased that to ~30% (Fig 10K). Treatment with SU5402 alone or SU5402 plus midi-GAGR did not increase the percent of dead cells (Fig 10K). Interestingly, pre-treatment with SU5402 significantly increased the percent of dead cells up to ~80% upon the post-treatment with 4HNE (Fig 10K). In neurons pre-treated with SU5402 and then treated with 4HNE, midi-GAGR could not decrease the percent of dead cells. This result indicates that FGFR1-mediated signaling pathway plays a major role in midi-GAGR-mediated neuroprotection against oxidative insult.

### Intranasally administered midi-GAGR increases neuronal activity markers and reduces hyperphosphorylated tau in 3xTg-AD mice

**Fig 11:**
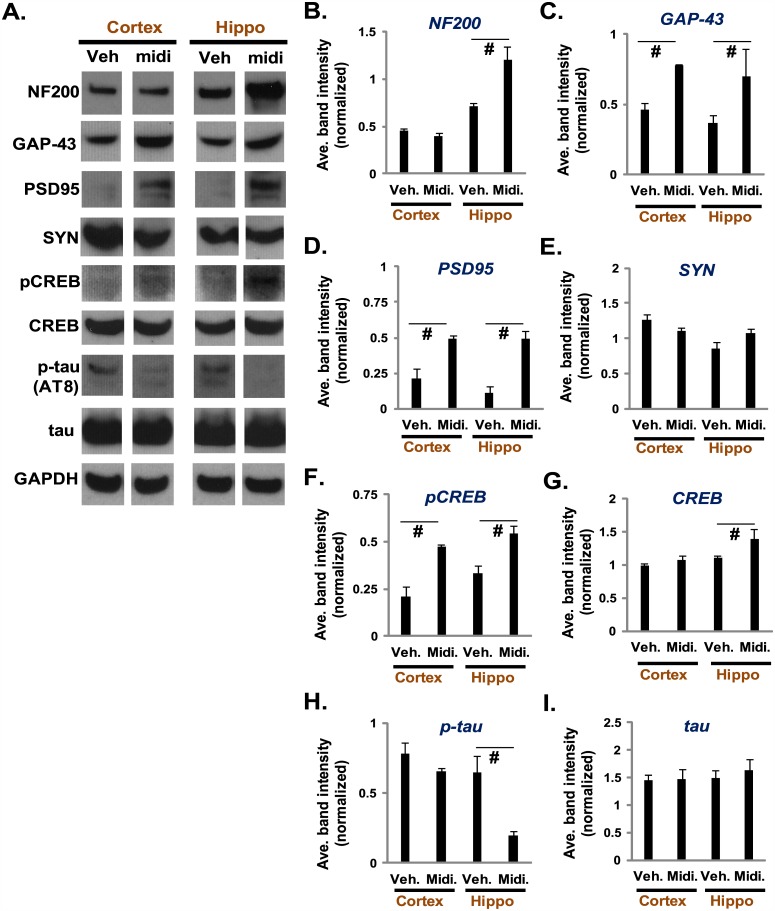
Intranasally administered midi-GAGR increased neuronal activity markers and decreased hyperphosphorylated tau in 3xTg-AD mice. (A) 12-week-old 3xTg-AD mice were intranasally administered with 40 μL (20 μL/nostril) of either sterile H_2_O (Veh.) or 1 mM midi-GAGR (midi) every day for 14 days and processed to obtain the cortex and hippocampus (Hippo). The tissues were homogenized to extract proteins for immunoblotting using the antibodies to NF200, GAP-43, PSD95, synaptophysin, pCREB, CREB, p-tau (AT8), tau, and GAPDH. (B-J) The densities of protein bands were measured using Image J and normalized to that of the loading control, GAPDH. Normalized values were used to calculate the average normalized band densities of NF200 (B), GAP-43 (C), PSD95 (D), synaptophysin (SYN, E), pCREB (F), total CREB (G), p-tau (H), and total tau (I). Data represent mean ± SEM of three independent animals. #, p<0.01, *, p<0.05.

Given that the activation of FGFR1 enhances neuronal activity and, possibly, attenuates neurodegeneration [51–53], we speculate that midi-GAGR treatment does the similar. Thus, we examined if midi-GAGR treatment increases the protein markers of neuronal activity and reduces the neurodegeneration marker, hyperphosphorylated tau (P-Ser202), in AD brain [102]. We used 12-month-old 3xTg-AD mice that harbor two familial AD mutations, APP_swe_ and PS1_M146V_, and the tau_P301L_ mutation found in frontotemporal dementia [103, 104]. Until 12 months of age, 3xTg-AD mice [103] develop synapse loss, Aβ peptide accumulation [105, 106], memory deficit [107], and tau hyperphosphorylation [103]. We used female 3xTg-AD mice since females show more obvious cognitive defects than males [108–110]. We administered 40 μL of vehicle (sterile H_2_O) or 1 mM midi-GAGR intranasally into female 3xTg-AD mice once per day for 14 days. During midi-GAGR administration, 3xTg-AD mice did not show any abnormal behavior. Of note, 3xTg-AD mice administered with midi-GAGR made tight nest every day while those with vehicle made loose nest as previously reported [111]. After 14-day administration, mice were killed for brain extraction. Extracted brains were dissected to obtain the cortices and hippocampi. The brain tissues were lysed in PMEE buffer containing 1% Igepal CA-630 and phosphatase and protease inhibitor cocktails for immunoblotting using antibodies to NF200, GAP-43, PSD95, synaptophysin (SYN), pCREB, CREB, p-tau (AT8), tau, and GAPDH. The protein band densities of detected proteins were normalized by those of GAPDH and used to calculate average normalized band density for each protein. Compared to 3xTg-AD mice administered with vehicle (Veh.), NF200 was increased significantly in the hippocampus of those with midi-GAGR while not changed in the cortex (Fig 11A and 11B). GAP-43 and PSD95, the postsynaptic markers for increased synaptic activity, were increased significantly in both cortex and hippocampus of 3xTg-AD mice treated with midi-GAGR compared to control while synaptophysin (SYN) remained unchanged (Fig 11A, 11C, 11D and 11E). pCREB, another postsynaptic marker that shows increased neurotrophic signaling, was also significantly increased in both cortices and hippocampi of 3xTg-AD mice treated with midi-GAGR compared to control (Fig 11A and 11F). Total CREB was slightly increased in the hippocampus of midi-GAGR-treated 3xTg-AD mice while not changed in the cortex (Fig 11A and 11G). Surprisingly, hyperphosphorylated tau (P-Ser202) was drastically decreased in the hippocampus of 3xTg-AD mice and slightly in their cortices (Fig 11A and 11H). Total tau was not changed (Fig 11A and 11I). These results indicate that intranasally administered midi-GAGR not only enhances neuronal and synaptic activities in the cortex and hippocampus but also decreased hyperphosphorylated tau, a major AD facilitator, in the brains of 3xTg-AD mice.

## Discussion

Neurotrophic treatments have shown a promise in slowing neurodegeneration [8, 13–21, 23] while their *in vivo* pre-clinical and clinical trials do not yield satisfactory outcomes yet. The poor outcomes are mainly due to the poor BBB-permeability and short plasma half-life of neurotrophic peptides [32, 33]. Later, cell graft, Trojan horse, and nanoparticle were developed for the delivery of neurotrophic peptides [34–39] but still limited by several problems such as the unregulated expression of neurotrophic peptide, the complication accompanied with multiple surgeries, and still the short plasma half-life of neurotrophic peptide. As such, no solution has been found to overcome the limits of neurotrophic peptides.

Prompted by the findings of neuroprotective polysaccharides [40–43], we searched for an alternative to neurotrophic peptide and finally discovered a 4.7kD neurotrophic polysaccharide that appears to overcome the limitations of neurotrophic peptides. In consistent with our previous study [101], intranasally administered midi-GAGR penetrates the BBB and exerts its neurotrophic effect for 24 h after one time administration. It suggests that midi-GAGR has a good BBB-permeability and a long plasma half-life of ~24 h. The latter long plasma half-life of midi-GAGR was expected as other polysaccharides show long plasma half-life as well [44–47]. Likewise, the good BBB-permeability of intranasally administered midi-GAGR is not surprising as the amino polysaccharide, chitosan, shows high BBB-permeability through intranasal administration [37–39, 48, 49]. Although intranasal administration has shown mediocre efficacy in delivering peptides through the BBB, non-peptide agents have shown success in their penetration into human brains via intranasal administration [112–115].

Given that midi-GAGR is derived from the human food additive, low acyl gellan gum (LA-GAGR), that has few side effects in human [50] and animals (FDA report), we speculate that midi-GAGR would not have serious side effect in human if clinically applied in the future. As such, ingested low acyl gellan gum that is expected to be digested to the size similar to that of midi-GAGR by natural cleavage does not show any side effects. Indeed, during our study, animals administered daily with midi-GAGR for two weeks did not show any abnormality in general behaviors such as moving, eating, sleeping, skin sensitivity, grouping, etc. Nevertheless, a possible toxicity of midi-GAGR should be examined in our next study. Meanwhile, one noticeable thing was that midi-GAGR-treated 3xTg-AD mice showed better nesting behavior than vehicle-treated. The nesting behavior is known to be impaired in 3xTg-AD mice, which corresponds to “deterioration of executive functions and daily live activities (DLA)” in human [111]. DLA is used along with the behavioral and psychological symptoms of dementia to diagnose early AD in human. Moreover, nesting behavior heavily depends on the hippocampus [116]. We believe that midi-GAGR may rescue some hippocampal function involved in nesting behavior. The nesting behavior of 3xTg-AD mice treated with midi-GAGR will be examined in our further study.

In our *in vitro* experiments, midi-GAGR showed a strong neuroprotective effect. At 0.1–1 μM, midi-GAGR protected the neurites of differentiated N2A cells up to 70–90% from the atrophy caused by the supra-physiological concentrations of two free reactive radicals, 4HNE and H_2_O_2_. At 1 μM, midi-GAGR protected primary rodent cortical neurons from the pathological concentrations of post-/co-treated free radicals and amyloid β_42_ peptide. The protective effect of midi-GAGR against co-existing free radicals and amyloid β_42_ peptide is clinically relevant as treatment is usually applied to the pre-existing pathological conditions like high levels of free radicals and amyloid β_42_ peptide. We also observed that 1 mM midi-GAGR protected rodent cortical neurons from more vicious neurodegenerative factor, activated microglial cells [96]. Thus, it is clear that midi-GAGR is a strong neuroprotective agent. In contrast, the control polysaccharides, dextran (D-Glc polymer), alginate (D-GlcA polymer), and HA-GAGR (highly acylated LA-GAGR), could not protect neurons from both free radicals and amyloid β_42_ peptide. Thus, the neuroprotective effect of midi-GAGR appears to be sequence-specific, which may confer midi-GAGR a specific binding to a certain molecule such as receptor.

In addition to the neuroprotective effect, midi-GAGR showed a strong neurotrophic effect both *in vitro* and *in vivo*. *In vitro*, midi-GAGR greatly enhanced neuritogenesis in N2A cells at 0.1–1 μM and in rodent cortical neurons at 1 μM. Other control polysaccharides such as dextran, alginate, and HA-GAGR could not enhance neuritogenesis. Similarly, in rodent cortical neurons, midi-GAGR increased the nuclear levels of activated pCREB, the transcriptional factor that enhances neuronal activities for survival and memory [117–119], while dextran and alginate did not. HA-GAGR appears to activate CREB to some extent but less than midi-GAGR. We observed a similar neurotrophic effect of midi-GAGR in the brains of rodents that were intranasally administered with midi-GAGR. In the frontal cortices and hippocampi of midi-GAGR-administered animals, the markers for neuritogenesis (NF200) and synaptic activity (GAP-43) were increased within 24 h after one-time intranasal spray. Thus, these *in vitro* and *in vivo* evidences clearly manifest the neurotrophic property of midi-GAGR.

Our next question was what is the neurotrophic mechanism used by midi-GAGR? To find an answer, we performed pulldown experiment and pharmacological inhibitor study and found that midi-GAGR interacts with FGFR1 and appears to activate two FGFR1 signaling pathways, FRS2a-Shc-Grb2-PKC-Raf-MEK and PI3K-Akt/PLCγ-Ca^2+^-CaMKII pathway [73, 75, 76]. Based on the observation that the levels of pCREB were reduced more drastically by PKC and MEK inhibitors than by PI3K and CaMKII, FRS2a-Shc-Grb2-PKC-Raf-MEK pathway plays a more major role than PI3K-Akt/PLCγ-Ca^2+^-CaMKII pathway for CREB activation. We also found that FAK is not involved in midi-GAGR-FGFR1 signaling pathway as midi-GAGR-induced CREB phosphorylation was not decreased by the inhibitor of FAK that sits at the bottleneck of NCAM180 signaling pathway [73]. In our opinion, the selective activation of FGFR1 over NCAM180 (possibly, other NCAMs, too) should be beneficial in the clinical application of midi-GAGR since the activation of NCAM often leads to undesired effects [120]. Meanwhile, it is unclear yet whether midi-GAGR binds to FGFR1 in the similar manner to either FGF or FGL. Nonetheless, both FGF (e.g., FGF2) and FGL have shown a great efficacy in attenuating neurodegeneration and improve memory in AD mice (FGF2 [60–68]; FGL [52, 79–83]). Moreover, both FGF2 and FGL reduce AD pathogenic factors (FGF2 [69, 70]; FGL [82]). Therefore, midi-GAGR may exert the similar beneficial effects to FGF or FGL while with longer plasma half-life than those peptides.

As most polysaccharides are good antioxidants, midi-GAGR and other control polysaccharides used in our study were expected to show some extents of protection of cells and neurons from free reactive radicals. However, at the low concentrations below 1 μM, dextran and alginate could not protect rodent cortical neurons from the pathological concentrations of 4HNE and H_2_O_2_. Moreover, the polysaccharides and HA-GAGR could not protect the neurons from the relatively low but pathological concentration (2 μM) of Aβ_42_ peptide. In contrast to the control polysaccharides, midi-GAGR as well as LA-GAGR could protect the neurons from all three neurodegenerative factors, 4HNE, H_2_O_2_, and Aβ_42_ peptide. This result indicates that the neuroprotective effect of midi-GAGR does not depend on its antioxidant property. To further clarify this, we pre-treated rodent cortical neurons with FGFR1 inhibitor prior to the treatment with 4HNE. Midi-GAGR could not rescue neurons from 4HNE-induced death in neurons pre-treated with FGFR1 inhibitor, suggesting that FGFR1-mediated neurotrophic signaling pathway is the major mechanism by which midi-GAGR rescues neurons from oxidative insult-induced death. What is more, FGFR1 inhibitor increased the percent of neuron death caused by 4HNE to even higher than vehicle plus 4HNE. This suggests that FGFR1 is essential for neuron survival in the presence of high oxidative insult.

As discussed thus far, midi-GAGR has a great therapeutic potential (neurotrophic, neuroprotective, BBB-permeable, and long plasma half-life) for the treatment of neurodegenerative diseases, especially AD. Thus, we examined briefly the efficacy of midi-GAGR in enhancing neuronal activity and reducing pathogenic factor in the brains of 3xTg-AD mice that are known to demonstrate most AD pathogenic progresses until the age of 12 month [121]. In consistent with the results of SD rats, intranasally administered midi-GAGR increased both NF200 and GAP-43, the markers of increased neuritogenesis and synaptic activity, respectively, in the hippocampus. In the cortex, only GAP-43 was increased while NF200 was not changed. No change in NF200 in the cortex may be due to the saturation of the neuritogenic effect of midi-GAGR at the concentration of equal to or higher than 1 μM similarly to the result of our *in vitro* experiment (Fig 6). In addition to NF200 and GAP-43, PSD95 and pCREB, the indicators of increased synaptic activity, were also increased in both cortex and hippocampus. The increase in all four markers for enhanced neuronal activity in the brain of 3xTg-AD mice is the undoubtful evidence that shows a great potential of midi-GAGR for restoring the learning and memory function of the brain in AD mice.

In addition to the increases in neuronal activity markers, we were surprised to observe the reduction of hyperphosphorylated tau (P-Ser202) in the hippocampi of 3xTg-AD mice. Hyperphosphorylated tau is a reliable biomarker for “mild cognitive impairment” (MCI) [122, 123] at early AD. Hyperphosphorylation impairs the function of tau in promoting microtubule polymerization, resulting in its aggregation to form neurofibrillary tangles (NFTs), microtubule disassembly, and loss of axonal microtubule-based transport [102, 124]. Given that the mechanism by which the hyperphosphoryation of tau is regulated is complicated [125–130], another intensive study is needed to address how midi-GAGR decreases tau hyperphosphorylation. Nonetheless, we speculate that midi-GAGR may inhibit GSK3β, the major kinase responsible for tau hyperphosphorylation, in a similar way to FGL [82].

## Conclusions

We discovered a BBB-permeable, long plasma half-life, strong neuroprotective and neurotrophic polysaccharide, midi-GAGR. Midi-GAGR is a small cleavage product of low acyl gellan gum that is an FDA-approved food additive that has few side effects in human. BBB-permeable midi-GAGR can exert its neurotrophic effect in the cortex and hippocampus for ~24 h after one-time intranasal administration. Moreover, midi-GAGR not only increases protein markers for increased neuronal activity but also reduces hyperphosphorylated tau in the brains of 3xTg-AD mice. To this end, we believe that all the great properties of midi-GAGR show its great potential as therapeutic agent for the treatment of neurodegenerative diseases especially AD. If clinically developed, midi-GAGR is expected to improve the neurotrophic treatment of neurodegenerative diseases.

## Supporting Information

S1 Appendix 24-h enzymatic digestion of low acyl gellan gum (LA-GAGR). The viscosity—storage modulus profiles showing the MWs of LA-GAGR (A) and its cleavage product after 24-h enzymatic hydrolysis (B).
(EPS)

S2 Appendix 48-h and 72-h enzymatic digestion of LA-GAGR. The viscosity—storage modulus profiles showing the MWs of the cleavage products of LA-GAGR after 48-h (A) and 72-h (B) enzymatic hydrolysis.
(EPS)

S3 Appendix Immunostaining of purified microglial cells with the antibody to Iba-1. Purified microglial cells were plated on glass coverslips coated with poly-L-lysine. After 24 h, microglial cells were fixed and immunostained with Iba-1 (microglial marker, green) and βIII-tubulin (neuron marker, red). Representative image shows microglial cells (green) and neurons (red). The percent of neurons in purified microglial cells was <2%.
(EPS)
